# Maximal cardiopulmonary exercise testing in laryngectomised patients using different heat and moisture exchangers – feasibility and exercise responses

**DOI:** 10.1017/S0022215123001068

**Published:** 2024-02

**Authors:** Anne N Heirman, Wim G Groen, Lisette van der Molen, Richard Dirven, Michiel W M van den Brekel, Martijn M Stuiver

**Affiliations:** 1 Department of Head and Neck Oncology and Surgery, Netherlands Cancer Institute, Amsterdam, the Netherlands; 2 Division of Psychosocial Research and Epidemiology, Netherlands Cancer Institute, Amsterdam, the Netherlands; 3 Amsterdam Center of Language and Communication, University of Amsterdam, Amsterdam, the Netherlands; 4 Center of Expertise Urban Vitality, Faculty of Health, Amsterdam University of Applied Sciences, Amsterdam, the Netherlands,; 5 Department of Epidemiology and Data Science, Amsterdam University Medical Centers, Amsterdam, the Netherlands

**Keywords:** Laryngectomy, exercise training, head and neck cancer

## Abstract

**Objective:**

After laryngectomy, the breathing resistance of heat and moisture exchangers may limit exercise capacity. Breathing gas analysis during cardiopulmonary exercise testing is not possible using regular masks. This study tested the feasibility of cardiopulmonary exercise testing with a heat and moisture exchanger in situ, using an in-house designed connector. Additionally, we explored the effect of different heat and moisture exchanger resistances on exercise capacity in this group.

**Methods:**

Ten participants underwent two cardiopulmonary exercise tests using their daily life heat and moisture exchanger (0.3 hPa or 0.6 hPa) and one specifically developed for activity (0.15 hPa). Heat and moisture exchanger order was randomised and blinded.

**Results:**

All participants completed both tests. No (serious) adverse events occurred. Only four subjects reached a respiratory exchange ratio of more than 1.1 in at least one test. Maximum exercise levels using heat and moisture exchangers with different resistances did not differ.

**Conclusion:**

Cardiopulmonary exercise testing in laryngectomees with a heat and moisture exchanger is feasible; however, the protocol does not seem appropriate to reach this group's maximal exercise capacity. Lowering heat and moisture exchanger resistance does not increase exercise capacity in this sample.

## Introduction

A total laryngectomy is performed as primary treatment for advanced stages of laryngeal and hypopharyngeal carcinomas or as a salvage treatment. As total laryngectomy separates the upper and lower respiratory tracts, air no longer passes through the upper airways. Instead, air is inhaled through a stoma in the neck, where it immediately enters the lower airways.^1,2^ The upper airway heats, humidifies and filters inhaled air. After a laryngectomy, these functions are lost. As a result, patients experience increased pulmonary symptoms, such as involuntary coughing, mucus retention and a need for forced expectoration to clear the airways.^3,4^ Additionally, patients have a higher risk of infection and inflammation of the airways.^5,6^ These airway problems significantly influence sleep, social contacts and quality of life.^7^

Stoma cloth covers (bibs) and heat and moisture exchangers have been developed to restore some of these lost functions. Bibs are worn in front of the stoma. They can provide a good level of heating and moisturising of the inhaled air when worn correctly, and are reusable and inexpensive.^8^ On the downside, bibs give difficulty in occluding the stoma to speak, have a high breathing resistance and are usually not preferred by patients. Therefore, heat and moisture exchangers are the preferred devices in most developed countries.^9^

Heat and moisture exchangers are positioned in an adhesive baseplate, which is placed over the patient's stoma or in a cannula. The device passively retains the heat and moisture from expired air, which is then transferred to the inhaled air of the next breath.^10–12^ Heat and moisture exchangers improve the tracheal climate, resulting in less involuntary coughing and less sputum production.^11^ Long-term use of heat and moisture exchangers has been shown to prevent and even restore the loss of tracheal ciliated cells, and to improve quality of life.^12^

The size of the heat and moisture exchanger, as well as the internal pore sizes and salt concentration, determine the performance and resistance of a heat and moisture exchanger.^13,14^ Heat and moisture exchangers worn in front of a stoma have limited space and size. As a consequence, there is always a compromise between the efficacy or performance of a heat and moisture exchanger and its resistance. Heat and moisture exchanger resistance has been reported as a limiting factor for compliance (continuous use of a heat and moisture exchanger), and might cause discomfort during physical activity when ventilation demand increases.^11^ Such discomfort contributes to lower levels of heat and moisture exchanger compliance, and may cause patients to avoid physical activity or exercise. This, in turn, can lead to poorer overall health and fitness, as well as a lower quality of life.^15^

There are multiple heat and moisture exchangers available, with different levels of resistance and humidification, aiming to serve specific purposes such as higher filtration of (polluted) air or enhancing suitability for physical activities. In general, it is accepted that better heat and moisture exchangers (with higher resistance) and high compliance (wearing them 24 hours a day, 7 days a week) have a positive effect on symptoms and quality of life.^16^ Aside from differences in experienced comfort, it is currently unknown to what extent differences in heat and moisture exchanger resistance influence acute exercise responses, and exercise capacity and performance.

In order to understand the differences between heat and moisture exchangers in terms of their impact on exercise tolerance and exercise response, exercise testing is required. The ‘gold standard’ for cardiopulmonary exercise testing is a maximal cardiopulmonary exercise test with breathing gas analysis.^17–19^ In patients who have undergone laryngectomy, this task is complicated, as the regular facial masks used for breathing gas analysis cannot be used. Previously, exercise testing using a custom-made adapter attached to the base plate of the heat and moisture exchanger, but without the heat and moisture exchanger being in place, proved to be feasible.^20,21^ However, this approach discards the effect of the heat and moisture exchanger on upper airway physiology. In addition, it creates an unnatural situation for patients, which may affect the results of exercise testing.

In order to enable research into the impact of a heat and moisture exchanger's resistance on exercise capacity, we developed an adapter that allows exercise testing with the heat and moisture exchanger in situ. In this exploratory study, we tested the feasibility of this set-up for cardiopulmonary exercise testing in 10 laryngectomised patients, and explored the influence of heat and moisture exchanger resistance on exercise capacity and performance.

## Materials and methods

### Participants

Ten subjects were included in the study, selected using convenience sampling from the out-patient pool of the Department of Head and Neck Oncology and Surgery at the Netherlands Cancer Institute. All patients had undergone a total laryngectomy at least six months previously. The inclusion criteria were: fully independent in activities of daily living, and regular self-reported participation in moderate-intensity physical leisure activities (more than 30 minutes), three or more times a week. A history of cardiac problems (such as unstable angina, arrhythmias, myocardial infarction, syncope)^22^ and active oncological disease were exclusion criteria. All patients were daily users of heat and moisture exchangers (Provox Life Home or Provox Life Go; Atos Medical, Hörby, Sweden).

### Heat and moisture exchangers

All subjects were tested twice to allow comparison of exercise capacity and performance between heat and moisture exchangers with different resistance properties. Patients used their regular heat and moisture exchanger, which could be either the Provox Life Home or Provox Life Go, in one of the tests, and a lower resistance heat and moisture exchanger: the Provox Life Energy heat and moisture exchanger (Atos Medical), specifically developed for use during physical activity, in the other test. Heat and moisture exchanger specifications for resistance and moisture loss of each of the heat and moisture exchangers used in the study can be seen in Table 1.

**Table 1.** HME specifications*

HME product type	Flow resistance	Moisture loss
Provox Life Energy	0.15 hPa	23.0 mg/l
Provox Life Go	0.30 hPa	22.5 mg/l
Provox Life Home	0.60 hPa	19.5 mg/l

* These technical data come from the instructions for use of the products. Flow resistance is measured as 30 l/minute (Pa). HME = heat and moisture exchanger

### Connector for cardiopulmonary exercise testing

**Figure 1.**
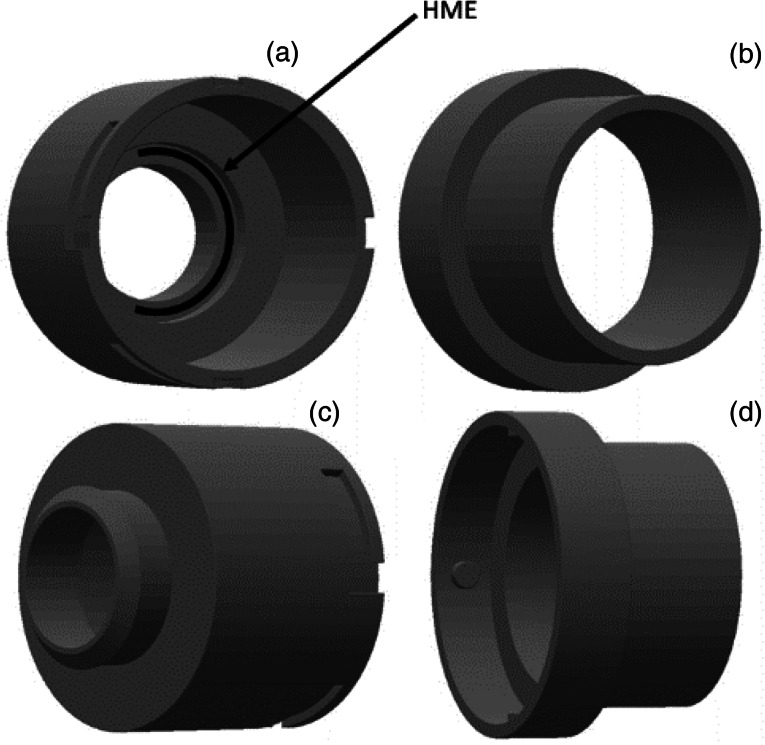
The three-dimensional printed connector. (a) The inside of the connector; the arrow shows where the heat and moisture exchanger (HME) is placed. (a & c) This part is connected to the patient's stoma by placing it into the adhesive baseplate. (b & d) This part is connected to the computer for breathing gas analysis. As visible in parts (c) and (d), the two parts can be connected by sliding and locking it.

An in-house three-dimensional (3D) printed adapter was developed to enable the replacement of the regular facial masks used for breathing gas analysis, which can be connected to the adhesive baseplate in front of the tracheostoma, with the heat and moisture exchanger in place. The connector was designed in such a way that the heat and moisture exchanger is not visible to the patient once placed and closed, enabling blinding during testing. The breathing gas analysis was corrected for the additional dead space of the connector (78.68 ml). Figure 1 gives an overview of the connector, and shows where the heat and moisture exchanger is placed in the connector. The connector is made out of polyamide 12 (Oceanz PA12; Oceanz 3D Printing, Ede, the Netherlands),^23^ and can be cleaned by washing it with water and soap and rinsing it with disinfectant (70 per cent alcohol).

### Study design

Participants were randomised into two groups (1:1) using opaque envelopes, which determined the order of the heat and moisture exchangers to be used during cardiopulmonary exercise testing. In the test, patients were blinded to the heat and moisture exchanger in use, which was installed in the 3D-printed connector (Figure 1) by the researcher administering the test (author ANH).

### Exercise testing protocol

Cardiopulmonary exercise tests were conducted on two separate visits, with two weeks in between. The tests were performed using an electronically braked cycle ergometer (Corival; Lode, Groningen, the Netherlands). Throughout testing, the subject's heart rate was continuously monitored with a 12-lead electrocardiogram (ECG). Breathing gas analysis was carried out with a calibrated ergospirometry system (Jaeger^™^ MasterScreen^™^ CPX). Immediately after finishing the test, participants were asked to rate their perceived level of exertion and dyspnoea, on a Borg scale ranging from 6 to 20, with 20 indicating maximal exertion.^24^

Prior to testing, there was a 5-minute period of slow pedalling, so that subjects could familiarise themselves with the bicycle. The test started with 3 minutes of unloaded cycling, followed by an increase in load every minute until subjects could no longer maintain the desired cadence (60–80 revolutions per minute). The load was increased by 15–20 W per minute, based on the participant's estimated fitness level.^25^ Patients were encouraged to push themselves maximally during the cardiopulmonary exercise testing. Indications for terminating cardiopulmonary exercise testing were: chest pain, ischaemic ECG changes, sudden pallor, signs of respiratory failure and the patient's wish to stop. Before the test, it was explained to patients that symptoms such as muscle fatigue and exhaustion are not reasons for stopping, but are normal responses to the effort on the cardiopulmonary exercise test.

The maximum rate of oxygen consumption (VO_2_ peak) during physical exertion was calculated by taking the average value over the last 30 seconds of the exercise test. Anaerobic threshold was determined using the V-slope method.^26^

**Figure 2.**
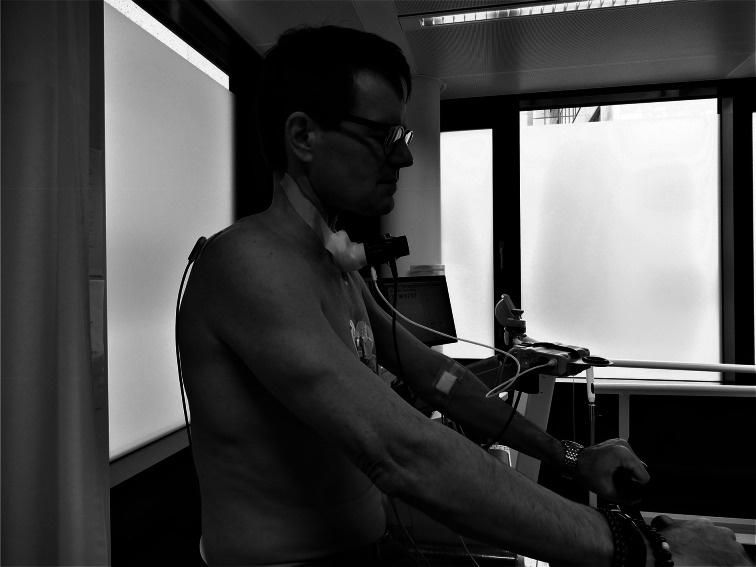
Cardiopulmonary exercise testing set-up. A study participant connected to a breathing gas analyser while seated on the exercise bicycle. Published with patient's permission.

The two exercise tests were conducted with the same test protocol and under similar testing conditions, with the only difference being the heat and moisture exchanger in use. Figure 2 shows the study set-up with a patient during cardiopulmonary exercise testing.

### Feasibility criteria

In order to explore the feasibility of this cardiopulmonary exercise testing set-up in laryngectomised patients, *a priori* criteria were set, based on literature and consensus in the research team. Specifically, each of the following points had to be satisfied. First, the connector was not leaking air during the measurements. Second, subjects were not complaining about discomfort using the connector with an adhesive baseplate and heat and moisture exchanger. Third, there was no occurrence of (serious) adverse events, such as hyperventilation, dyspnoea, ECG abnormalities, collapse, and so on. Fourth, at least 80 per cent of the patients were able to reach a respiratory exchange ratio of more than 1.1 (respiratory exchange ratio = metabolic production of carbon dioxide (VCO_2_) / uptake of oxygen (VO_2_)) and/or 95 per cent of their predicted maximum heart rate in at least one of the tests. (Ninety-five per cent predicted maximum heart rate formula, in beats per minute = (211 − 0.64 × age) × 95 per cent.) ^27,28^

### Cardiopulmonary exercise test data

Variables of interest were: time spent on the test; peak workload; heart rate (beats per minute); perceived level of exertion (Borg score;^24^ scores range from 6 to 20, with higher scores reflecting higher perceived effort); peak oxygen uptake (VO_2_ peak), both absolute (l/minute) and relative to body weight (ml/kg/minute); respiratory exchange ratio; anaerobic threshold (VO_2_/kg); and ventilatory efficiency, expressed as minute ventilation/carbon dioxide production at anaerobic threshold (VE/VCO_2_).

All data were calculated for the total group as well as for the subgroups based on patients’ regularly used heat and moisture exchanger type (0.3 hPa or 0.6 hPa), and the heat and moisture exchanger used during the test (regular or lowest resistance). We used graphs to plot outcomes for the 0.15 hPa heat and moisture exchanger (Provox Life Energy) against those obtained with patients’ regular (Provox Life Go or Provox Life Home) heat and moisture exchanger.

### Statistical analysis

All data were analysed using IBM® SPSS® Statistics software version 27 and GraphPad Prism 9 (GraphPad Software, San Diego, California, USA). As this was an exploratory study with a small sample size, we did not statistically test the differences for significance, but rather provide descriptive analyses and visual displays of patient data.

### Ethical considerations

The authors declare that all procedures contributing to this work are performed in accordance with the ethical standards of the medical ethical review committee of the Netherlands Cancer Institute, who approved the study (registration number: NL72840.031.20), and with the Helsinki Declaration of 1975, as revised in 2008.

## Results

### Subjects

Nine (out of 10) patients were male, and six of them had been treated with (chemo)radiotherapy before total laryngectomy. One participant had asthma, which was not limiting in terms of daily life activities and sports. None of the other participants were diagnosed with pulmonary diseases. All patients were former smokers but had stopped since treatment. Patient and treatment characteristics are shown in Table 2.

**Table 2.** Patient and treatment characteristics*

Characteristic	Values
Age (mean (range); years)	63.6 (49–79)
Gender (*n*)	
– Male	9
– Female	1
Time since total laryngectomy (mean (range); years)	10.7 (1–30)
Primary tumour (*n*)	
– Larynx	9
– Hypopharynx	1
Prior treatment (*n*)	
– RT/CRT	5/1
– Vertical hemi-laryngectomy	1
– None	3
Adjuvant treatment (*n*)	
– RT	2
– None	8
Neck dissection (*n*)	
– Bilateral	5
– None	5
Flap reconstruction (*n*)	
– Pectoralis major flap	2
– Supraclavicular artery island flap	1
– None or unknown^†^	7
Pulmonary disease(s) (*n*)	
– None	8
– Asthma	1
– Pulmonary embolism^‡^	1
Smoking history (*n*)	
– Former smoker	10
– Never smoker	0
Type of HME used daily (*n*)	
– Provox Life Home (0.6 hPa)	8
– Provox Life Go (0.3 hPa)	2

* Patients *n* = 10. ^†^No report available. ^‡^Pulmonary embolism in 2012. RT = radiotherapy; CRT = chemo-radiotherapy; HME = heat and moisture exchanger

### Feasibility of cardiopulmonary exercise testing

None of the 10 patients experienced air leakage through the connector and/or adhesive baseplate; there were no abnormalities in the results from the breathing gas analysis. All patients were comfortable wearing the connector. There were no complaints of discomfort or dyspnoea, and no (serious) adverse events occurred during the cardiopulmonary exercise tests or in the following days. Thus, all of the first three feasibility criteria were met. Feasibility criterion four was not met. A respiratory exchange ratio of more than 1.1 was reached by four patients, of whom two were in the first test and two were in the second (all with the 0.6 hPa heat and moisture exchanger), while only two patients reached this ratio in both tests. Only one subject reached 95 per cent of his maximum predicted heart rate in one test. This was the test with his regular 0.6 hPa heat and moisture exchanger (Tables 3 and 4).

**Table 3.** Median values at end of cardiopulmonary exercise testing

Parameter	Regular resistance (0.3 hPa or 0.6 hPa)			Lower resistance (0.15 hPa)		
	Total group	Usual HME type 0.6 hPa	Usual HME type 0.3 hPa	Total group	Usual HME type 0.6 hPa	Usual HME type 0.3 hPa
Cases (*n*)	10	8	2	10	8	2
Time on test (seconds)	1000	1006	930	999	1000	916
Peak workload (W)	141.2	148.1	101.0	129.7	136.2	90.0
HR (bpm)	138	138	134	133	133	131
Absolute VO_2_ peak (l/minute)	1.48	1.62	1.04	1.59	1.72	1.14
Relative VO_2_ peak (ml/kg/minute)	20.4	21.4	15.7	20.6	21.9	15.8
VE peak (l/minute)	57.1	61.2	42.0	53.0	56.2	45.0
Respiratory exchange ratio	1.05	1.10	0.85	1.00	1.05	0.91
Anaerobic threshold (VO_2_/kg)*	12.4 (*n* = 7)	12.0 (*n* = 6)	17.6 (*n* = 1)	12.0 (*n* = 7)	11.4 (*n* = 6)	13.6 (*n* = 1)
VE/VCO_2_ at anaerobic threshold	32.4	31.9	38.7	33.2	32.4	34.2
Borg score^24†^	15	15	16	14	14	16.5

*Anaerobic threshold was not reached by one subject and could not be determined in two subjects (see Table 4 for details). ^†^Minimum score of 6 = low effort; maximum score of 20 = high effort. HME = heat and moisture exchanger; HR = heart rate; bpm = beats per minute; VO_2_ = oxygen consumption; VE = minute ventilation; VE/VCO_2_ = minute ventilation/carbon dioxide production

**Table 4.** Individual results of cardiopulmonary exercise testing

Pt no.	RER – regular resistance		Peak workload (W)	VO_2_ peak (l/min)*	HR (bpm)	Reason for terminating exercise test	RER – lower resistance (0.15 hPa)	Peak workload (W)	VO_2_ peak (l/min)*	HR (bpm)	Reason for terminating exercise test
	0.3 hPa	0.6 hPa									
1	n/a	0.96	99	1.11	114	Leg fatigue, out of breath	1.03	93	1.19	119	Coughing & dyspnoea
2	n/a	1.06	129	1.62	136	Exhaustion	0.90	120	1.49	134	Exhaustion
3^†^	0.73	n/a	75	1.07	114	Leg fatigue, out of breath	0.79	87	1.14	115	Unwell because of emotional complaints due to unrelated life event
4	n/a	1.27^‡^	254	2.71	171**	Exhaustion	1.26^‡^	246	2.48	167	Exhaustion
5	n/a	1.16^‡^	170	1.88	127	Exhaustion	1.16^‡^	170	1.85	127	Exhaustion
6	n/a	1.10^‡^	108	1.33	141	Exhaustion	1.03	102	1.45	133	Leg muscle cramps
7	1.04	n/a	126	1.36	150	Dyspnoea, exhaustion	0.94	123	1.42	147	Leg fatigue & cramps, exhaustion
8^§^	n/a	1.06	69	1.31	148	Out of breath, exhaustion	0.95	60	1.14	127	Out of breath, exhaustion
9^§^	n/a	0.98	150	1.96	133	Leg fatigue, out of breath	1.06	132	1.66	113	Dyspnoea
10	n/a	1.15^‡^	159	1.80	146	Exhaustion	1.09	162	2.03	154	Exhaustion

*The highest achieved measured oxygen consumption (VO_2_) in this test. ^†^Did not reach anaerobic threshold. ^‡^Indicates respiratory exchange ratios (RERs) of more than 1.1. **Indicates when 95 per cent predicted heart rate (HR) is reached (calculated based on gender, age, weight and height). ^§^Anaerobic threshold could not be determined through V-slope method.^26^ Pt no. = patient number; min = minute; bpm = beats per minute; n/a = not applicable

### Overall cardiopulmonary exercise test results

The overall average time spent on the test was 958.5 seconds, with a median peak workload of 126 W (range, 39–254 W), and a median peak oxygen uptake (VO_2_ peak) of 1.55 l/minute (range, 1.07–2.71 l/minute), for all cardiopulmonary exercise tests conducted. Median peak oxygen uptake (VO_2_ peak) for patients reaching the respiratory exchange ratio of more than 1.1 was 1.85 l/minute (range, 1.33–2.71 l/minute). Under all testing conditions (Table 3), we observed normal responses to exercise for heart rate, oxygen consumption (VO_2_), ventilatory efficiency (minute ventilation/carbon dioxide production at anaerobic threshold; VE/VCO_2_), minute ventilation (VE) and respiratory exchange ratio. On average, relative VO_2_ peak was quite low, especially for the regular 0.3 hPa heat and moisture exchanger users. Regular 0.6 hPa heat and moisture exchanger users (*n* = 8) performed better compared to regular 0.3 hPa heat and moisture exchanger users (*n* = 2) in this sample: they cycled longer, achieved higher workloads and had higher peak values. When comparing the cardiopulmonary exercise test results obtained with the regular heat and moisture exchanger to those obtained using the lowest resistance heat and moisture exchanger, we observed no meaningful differences.

For seven patients, their anaerobic threshold could be determined (Table 4). For two patients (numbers 8 and 9), the anaerobic threshold could not be determined, while one subject (number 3) did not reach the anaerobic threshold in both tests because of subjective exhaustion and feeling out of breath. Only one subject reached his 95 per cent predicted heart rate, in the test with his regular resistance (0.6 hPa).

### Peak workload and peak oxygen consumption related to resistance level

**Figure 3.**
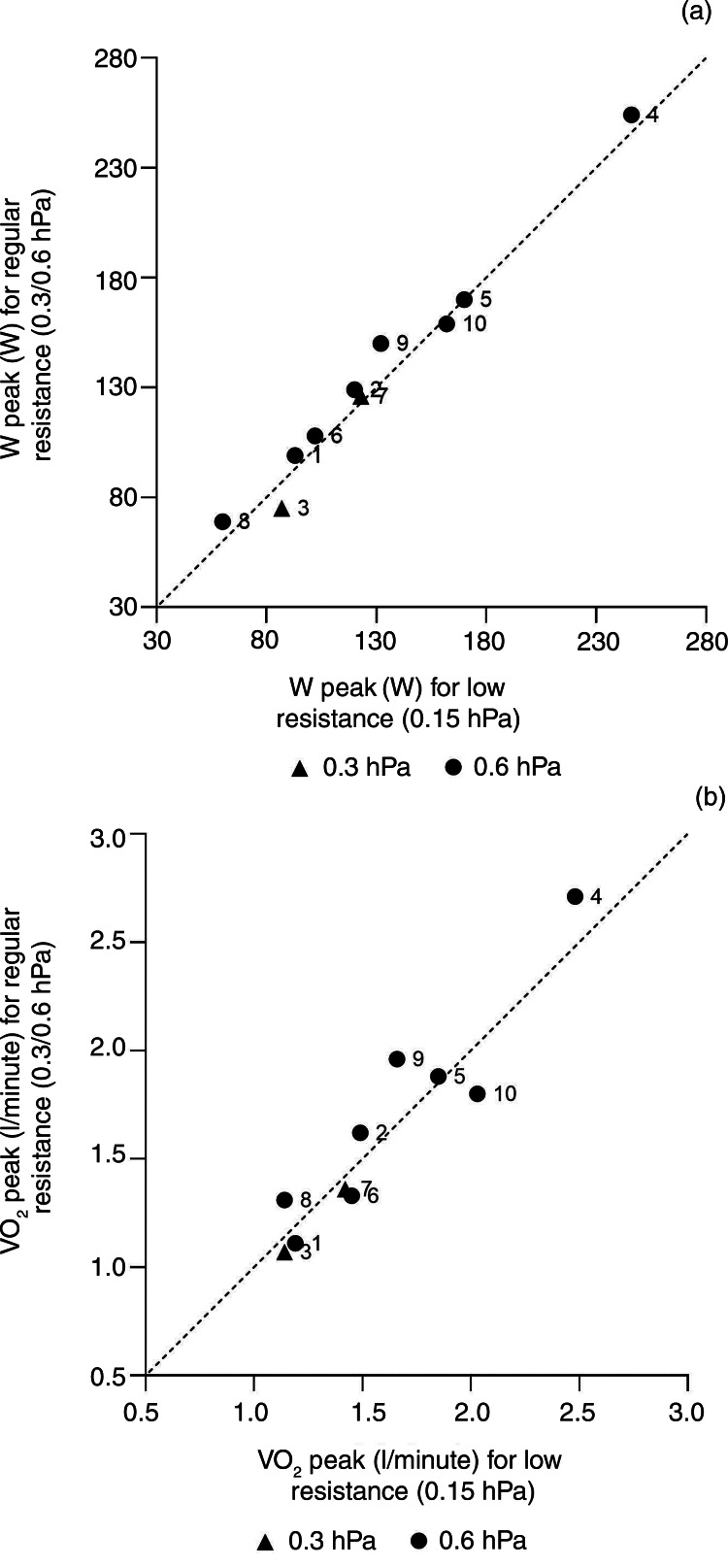
Intra-subject comparison for peak workload (W peak) (a) and peak oxygen uptake (VO_2_ peak) (b) between the different heat and moisture exchanger resistances. Regular heat and moisture exchanger resistance (of 0.3 hPa or 0.6 hPa) is compared to lower resistance (0.15 hPa). The dashed line is the identity line.

Figure 3 shows the comparisons of individual results per test. As can be seen from the graphs, patients performed similarly in both tests, regardless of the heat and moisture exchanger used. The results for peak oxygen consumption (VO_2_ peak, Figure 1b) show more variability between the two resistances compared to other outcomes, but these are still quite similar and within the limits of regular test–retest variability.^29^

## Discussion

Cardiopulmonary exercise testing with gas exchange analysis is possible in laryngectomy patients wearing a heat and moisture exchanger. There was no air leakage during testing, all patients were comfortable wearing the connector during their cardiopulmonary exercise tests, and there were no (serious) adverse events. However, fewer than half (4 of 10) of the patients reached a respiratory exchange ratio of more than 1.1 in at least one test, and only one subject reached 95 per cent of his predicted heart rate during one test. Thus, although this set-up of cardiopulmonary exercise testing is technically feasible and appears to be safe, it could be questioned whether the ramp protocol used is suitable for reaching maximum exercise capacity in this population.

The subjects’ reasons for terminating the cardiopulmonary exercise testing before reaching predicted maximum levels were mainly exhaustion, feeling out of breath and leg fatigue, without signs of cardiopulmonary problems. Instead, early stopping was related to a low subjective exercise tolerance.^30^ It is common that inactive patients do not tolerate normal acute physical responses induced by exercising. Improved tolerance can be achieved through training, which has proven to be effective in terms of properly performing cardiopulmonary exercise testing.^31^ Although we purposefully sampled our participants based on regular physical activity of at least moderate intensity, most participants habitually participated in predominantly low-intensity activities such as cycling (some with electric assistance), moderate strength training and walking. When selecting participants and determining the steps of the ramp protocol, we relied on self-reported levels of physical activity. However, in survivors of head and neck cancer, perceived levels of activity and fitness may not accurately reflect their actual levels.^32^ This should be considered when determining the ramp steps in future testing.

Another explanation for patients stopping because of feelings of exhaustion and leg fatigue could be that exercise capacity was limited by muscle strength, rather than aerobic capacity. Low skeletal muscle mass, sarcopenia, is relatively common (30–50 per cent) in patients who have undergone or are undergoing laryngectomy.^33,34^ Sarcopenia results in less muscle strength, affects gait, endurance and mobility, and may lead to inactivity that further impacts muscle mass and strength.^35^ Limited muscle strength after laryngectomy could be a problem when using the current cardiopulmonary exercise testing protocols, which also calls for smaller increments.

Only one participant reached their maximum predicted heart rate once. Although this could be due to peripheral fatigue limiting the test, overestimation of maximum predicted heart rate is common in low-fit subjects, and might be an alternative explanation for this finding.^36,37^

Similar results to ours were found in a group of haemato-oncological patients during treatment: cardiopulmonary exercise testing (without breathing gas analysis) was found to be feasible and safe, but only a minority of patients reached maximal effort.^28^ The researchers concluded that the protocol used might not be fitting for this low-fit and vulnerable group, and suggested the use of an endurance protocol at a fixed workload as a possible alternative. Although fixed-workload tests cannot be used to determine peak oxygen consumption (VO_2_ peak), they can be useful to evaluate changes in exercise capacity over time in individuals, and to assess intervention effects in comparative studies. The advantage of our current set-up is that it still enables breath gas analysis during such submaximal testing. As suggested by others, it would be an option to first train patients, before applying maximal cardiopulmonary exercise testing. For this purpose, muscle strength training, as well as aerobic training, have been suggested.^28,38–40^

In the context of rehabilitation, the question remains whether reaching maximal capacity should be a testing goal, or whether individual testing goals should be set to match the patient's treatment goals, wishes and possibilities. Further research is warranted to determine the best approach to exercise testing in low-fit cancer survivors, including those who have undergone total laryngectomy.

In those patients who reached a respiratory exchange ratio of more than 1.1, peak oxygen consumption (VO_2_ peak) relative to body weight was quite low (mean of 19.3 ml/kg/minute), especially considering their self-reported level of regular physical activity. As a reference, the minimum level of maximal oxygen uptake compatible with continued independence, relative to body weight, is about 15–18 ml/kg/minute, normally reached at 80–85 years in sedentary older adults.^38^

In this study, participants using the 0.3 hPa heat and moisture exchanger as a regular heat and moisture exchanger had poorer exercise performance than those using the 0.6 hPa heat and moisture exchanger. This is not likely the result of the heat and moisture exchanger used, but probably reflects that patients with a poorer general health or pulmonary condition choose a lower resistance heat and moisture exchanger for comfort.

- Cardiopulmonary exercise testing with breathing gas analysis with a heat and moisture exchanger seems safe and feasible in laryngectomised patients
- A specially designed connector is needed for gas analysis with a heat and moisture exchanger
- Lowering breathing resistance through a different type of heat and moisture exchanger was not beneficial for exercise capacity in our sample
- Laryngectomised patients may not be able to reach their maximum capacity during regular cardiopulmonary exercise testing protocols

We found that lowering the heat and moisture exchanger resistance level did not lead to improved exercise capacity. Non-laryngectomised people tend to lower the breathing resistance during exercise by mouth breathing. Therefore, we anticipated that a lower resistance would increase exercise capacity, but our data indicate otherwise. Of note, the resistance of the 0.6 hPa heat and moisture exchanger is still lower than that of nose breathing (1.9–3.9 hPa).^41^ Those who are comfortable wearing a regular heat and moisture exchanger under non-exercise conditions will not likely benefit from a lower resistance heat and moisture exchanger during exercise. In addition, they must be aware that using low-resistance heat and moisture exchangers may feel more comfortable, but comes at the cost of reduced health benefits associated with poorer performance of the heat and moisture exchanger in terms of heat and moisture retainment, and reduced respiratory muscle response to exercise training stimuli.

### Limitations

This is a small, randomised and single-blinded exploratory study, in which only self-reported active, predominantly male, laryngectomised patients have been included. Not blinding the researcher administering the test to the heat and moisture exchanger in use could have introduced performance bias. However, maximal encouragement was part of the standard operating procedure of the cardiopulmonary exercise testing. In addition, if a Pygmalion effect has occurred, this would likely have biased the findings towards better achievement using the lower resistance heat and moisture exchanger. As we did not observe a difference, we deem such bias unlikely. The results should not be extrapolated to the larger population of laryngectomised patients. A comparison of the influence of regular heat and moisture exchanger resistance on exercise performance was beyond the scope of the study, and so there is no equal distribution of the two types of regular heat and moisture exchangers.

As a pilot study, the study was not powered for the statistical testing of differences. Hence, our findings and interpretations should be considered with caution. Lung function tests were not performed prior to cardiopulmonary exercise testing. In hindsight, this would have been useful to obtain a better assessment of pulmonary function as a potentially limiting factor.

## Conclusion

This first exploratory study indicates that exercise testing using breathing gas analysis with a heat and moisture exchanger in situ is safe and technically feasible in laryngectomised patients. The conventional ramp protocol may not be ideal for reaching maximum exercise capacity in this vulnerable group. We found no indications that lowering the heat and moisture exchanger resistance improves exercise capacity in this study.
